# Low-Temperature Characteristics of Nanowire Network Demultiplexer for Qubit Biasing

**DOI:** 10.1021/acs.nanolett.1c04971

**Published:** 2022-05-12

**Authors:** Lasse Södergren, Patrik Olausson, Erik Lind

**Affiliations:** † Department of Electrical and Information Technology, Lund University, Box 118, SE-221 00 Lund, Sweden; ‡ NanoLund, Lund University, Box 118, SE-221 00 Lund, Sweden

**Keywords:** nanowire, multiplexer, InGaAs, cryogenic

## Abstract

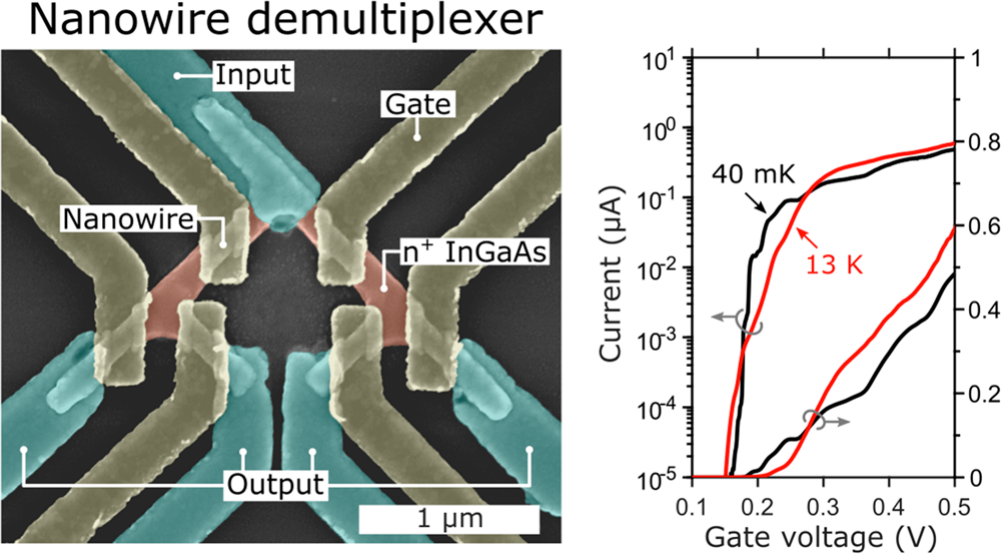

In current quantum computers, most qubit control electronics are connected to the qubit chip inside the cryostat by cables at room temperature. This poses a challenge when scaling the quantum chip to an increasing number of qubits. We present a lateral nanowire network 1-to-4 demultiplexer design fabricated by selective area grown InGaAs on InP, suitable for on chip routing of DC current for qubit biasing. We have characterized the device at cryogenic temperatures, and at 40 mK the device exhibits a minimum inverse subthreshold slope of 2 mV/dec, which is encouraging for low power operation. At low drain bias, the transmission breaks up into several resonance peaks due to a rough conduction band edge; this is qualitatively explained by a simple model based on a 1D real space tight-binding nonequilibrium Green’s functions model.

In current quantum computers, most low frequency bias, high frequency readout, and control electronics are generated at room temperature and connected with cables to the qubit chip at low temperatures. However, as quantum computers are scaled to a large number of qubits, the number of input/output (I/O) connections of the cryostat becomes unmanageable.^1−4^ By moving some of these circuits into the cryostat and operating them at cryogenic temperatures the amount of I/O needed can be reduced significantly. Therefore, there is a need for an increased effort in characterization of low-temperature electronics which are designed to operate at the low-temperature stages in scaled quantum computers. One circuit element of interest is the demultiplexing device for individually biasing many qubits with few input signals. Different kind of qubits such as transmons, spin qubits, or Majorana-based qubits all need current/voltage for biasing or control.^5−9^ For example, transmons need a biasing magnetic field provided by a current in close proximity on the qubit chip. Apart from the standard CMOS-implementation, multiplexing structures have also been demonstrated before in both III–V modulation doped quantum well devices^1^ and Si nanowires.^10^ In this paper, we have built a 1-to-4 demultiplexer (demux) proof of concept device based on a selective area grown lateral InGaAs nanowire network on InP with current control through gates coupled to the channel using high-κ oxide. Such a device can allow for highly scaled routing of bias currents/voltages, while operating under low power constraints due to a high on/off ratio and low on-resistance. The higher electron mobility and lower effective mass of the InGaAs nanowires compared to Si enables operation with lower drive voltage, which reduces the power dissipation, which is important for mK cryogenic operation where the cooling power is limited.^3^ On-chip (de)multiplexing devices can also be used to obtain statistics for wire-bonded devices operated in dilution fridges, where the device pin count can be limited.^11^ The device characteristics have been measured down to 40 mK. At cryogenic temperature the low inverse subthreshold slope allows the device to be efficiently turned off, minimizing the leakage currents. Operation of the fabricated devices at frequencies relevant for qubit control (∼1–5 GHz) requires some further considerations. The individual InGaAs nanowire transistors can be operated at high frequencies by utilizing a T-gate design which reduces parasitic capacitances and small gate lengths enabling a high transconductance.^12^ Considerations regarding the (de)multiplexer gate design also must be done to limit the capacitive leakage through the gates at the operating frequency.

A demultiplexer is a simple circuit which is able to deliver an input signal to one of many outputs. Figure 1a shows a schematic of a 1-to-4 demultiplexer; this network has one input at the source contact and four outputs at the drains. The gates (A, B) are used for controlling which drain the signal reaches. This type of network is very scalable, each added layer doubles the number of outputs while only requiring two more control signals, a 1:2^*n*^ multiplexer requires 2*n* control gates. Thus, the fabricated nanowire demultiplexing network shown in Figure 1b has four control gates (A_1_, A_2_, B_1_, B_2_) and four outputs drains (D_11_, D_12_, D_21_, D_22_). The proposed technology can be used to implement multiplexers with bigger fan-out (*n* > 2); this however requires a back-end-of-line technology for routing some of the gate wires as they need to cross some internal electrodes.^1^

**Figure 1:**
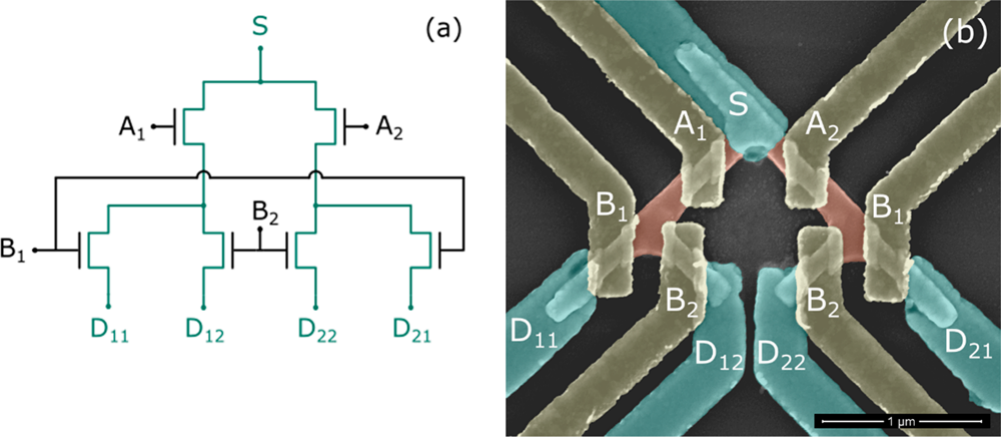
(a) A 1-to-4 demultiplexer circuit schematic with one input (S), four outputs (D) and four control gates (A, B). (b) False-colored scanning electron microscope (SEM) image of a demultiplexer. Source/drain contacts in blue, doped InGaAs in red, and gate contacts in yellow. The device has in total four different gates, A_1_, A_2_, B_1_, and B_2_, since the respective B gates are shorted (not shown). The unintentionally doped InGaAs channel, which can be seen under the gates have dimensions of *W* = 80 nm and *L*_G_ = 120 nm.

The lateral InGaAs nanowire network was fabricated on a semi-insulating InP:Fe (100) substrate by selective area epitaxy (SAE) using a metal organic vapor phase epitaxy (MOVPE) system. Figure 2 illustrates the fabrication process. Hydrogen silesquioxane (HSQ) patterned by an electron beam lithography (EBL) system was used as a growth mask. The mask openings were 80 nm wide and aligned to ⟨001⟩. This leads to nanowires defined by {110} facet sidewalls and (100) top surface, which limits the overgrowth of the mask.^13^ The sample was cleaned in 0.05% HF solution just prior to the first growth step consisting of 4 nm InP followed by 13 nm In_0.65_Ga_0.35_As grown at 600 °C. The increased indium content relative to the lattice matched (53% indium) gives a crystal with increased electron mobility, simultaneously the thickness of 13 nm InGaAs is not sufficient for relaxation to occur. Buffered oxide etch (BOE) was used to remove the HSQ mask before dummy gate HSQ lines were patterned along the (110) direction, defining the gate length of 120 nm. In the second growth step, 25 nm of doped In_0.65_Ga_0.35_As (*N*_D_ ≈ 5 × 10^19^ cm^–3^) contact layer was grown, which is sufficient to create a good contact with source/drain metal. After the HSQ was removed by BOE, a HSQ etch mask was patterned, outlining the mesa. Mesa isolation was done by wet etching the grown InGaAs using H_3_PO_4_/H_2_O_2_/H_2_O followed by a short HCl/H_2_O dip, etching 16 nm into the substrate. Plasma-enhanced chemical vapor deposition (PECVD) was used to deposit 14 nm of SiN_*x*_ before removing the HSQ with BOE, leaving SiN_*x*_ everywhere on the sample surface except the active device area. This ensures proper isolation between measurement pads. The surface of the sample is etched by ozone oxidation followed by diluted HCl. An EBL lift-off process and electron beam evaporation of Ti/Pd/Au was used to fabricate the source and drain contacts. Next, the surface was passivated by ozone cleaning and 20 min in (NH4)_2_S (10% solution in water) before deposition of Al_2_O_3_/HfO_2_ gate oxide (7/100 cycles) at 300/120 °C. The device is completed by gate definition by evaporation of Ti/Pd/Au using a lift-off process followed by a forming gas (H_2_/N_2_, 5/95%) annealed at 300 °C. The implemented structure thus allows routing of a current between input and output and avoids losses due to ohmic contacts within the structure. Other circuit elements, such as MOSFETs, varactors, and MIM-capacitors, can also be implemented within this material platform.

**Figure 2:**
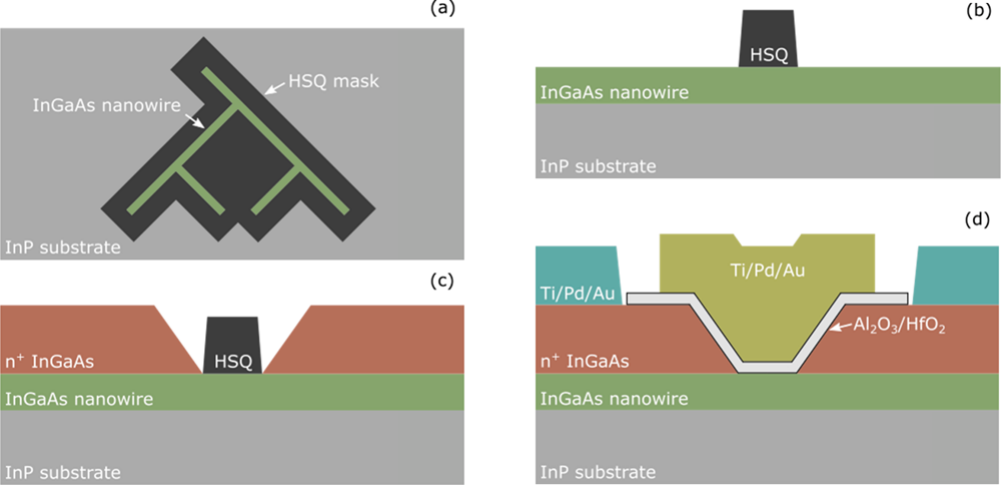
Simplified schematics of the fabrication steps for one gated region. (a) The InGaAs nanowire network is grown using selective area epitaxy with HSQ as a growth mask on an InP substrate. (b) An EBL defined HSQ dummy gate patterned across the nanowire. (c) Highly doped n^+^ InGaAs contacts are grown. (d) Final device structure after dummy gate removal, gate oxide deposition, and metallization steps.

The typical transfer characteristics at *V*_DS_ = 50 mV and 500 mV of a *W* = 80 nm and *L*_G_ = 120 nm demux device are presented in Figure 3. The measurements were done at 13 K by first simultaneously applying a constant drain bias to all of the drains; then, the current was sequentially routed to the respective drain. This was done by setting the respective gate A at a constant high voltage (∼800 mV), making sure the channel is in the on-state, then sweeping the voltage of the respective gate B. The two other gates are set in the off state. Doing the reverse, sweeping gate A while gate B is at a high voltage, yields similar characteristics. For example, to route the current between S and D_11_, gate A_1_ and B_1_ are turned on while gate A_2_ and B_2_ are turned off. All gate currents and the drain currents in the turned off paths are measured simultaneously to be below the noise floor of the measurement setup (<1 pA), demonstrating demultiplexing functionality. This demonstrates a well-behaved device with a very small gate leakage and good isolation between all gates and drains. The small signal transconductance of the devices with respect to a single gate is g_m_ ≈ 0.6 mS/μm.

**Figure 3:**
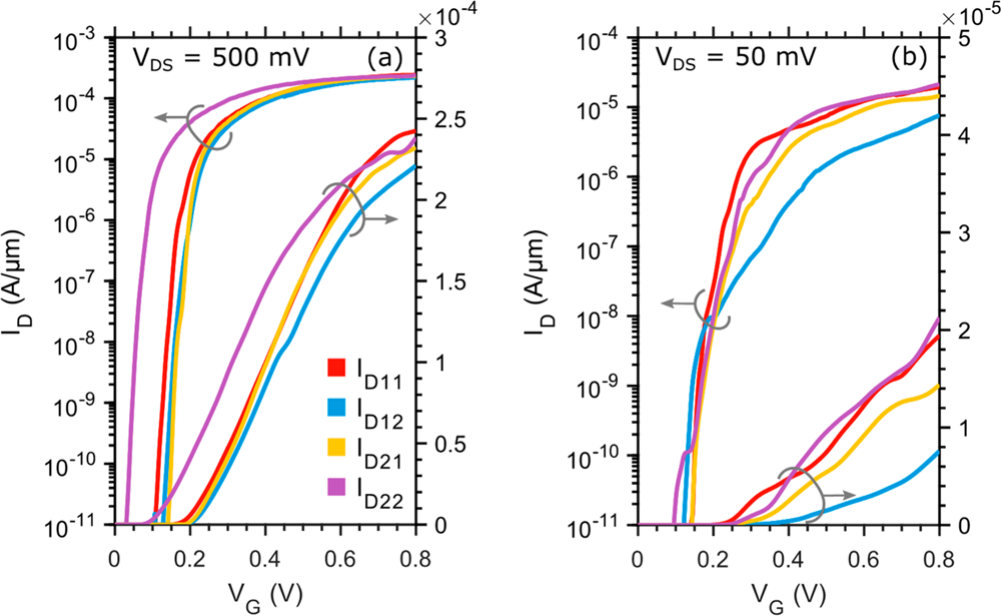
Low-temperature transfer characteristics normalized to the nanowire width at (a) *V*_DS_ = 500 mV and (b) *V*_DS_ = 50 mV measured at 13 K. The different colored lines refer to the drain current in each respective drain, as indicated in Figure 1. During the measurements, the drain bias was applied to all drains simultaneously, and the gates were then used to only set one source-drain path in a low resistive state at any given time. The current in the high resistive paths were below the noise floor of the measurement setup (<1 pA).

Statistics of device threshold voltages, minimum inverse subthreshold slopes, and on-resistances from several different devices are shown in Figure 4. The data is extracted from 8 demux devices, which essentially is equivalent to 32 available current paths, since there should be no difference between the 4 drains. The difference in the median threshold voltage is within 100 mV between the four drains. The total median minimum inverse subthreshold slope of all current paths is below 10 mV/dec, showing a good control of the channel electrostatics. The on-resistance is extracted at 300 mV above *V*_T_ with a total median of 6800 Ωμm. Measurements on similar samples show that the metal/semiconductor contact resistance *R*_C_ = 20 Ωμm, and the access resistance through the doped n+ InGaAs layer *R*_A_ = 30 Ωμm are both reasonably low. This suggests there is some additional series resistance in the structure, most likely originating from the interface between the undoped InGaAs channel and the doped n+ InGaAs layer. Although the statistics are limited, this data indicates a high process yield with all demultiplexers operational. Only one source-drain current path exhibits high *V*_T_ and *R*_on_, resulting in an effective yield of over 95%.

**Figure 4:**
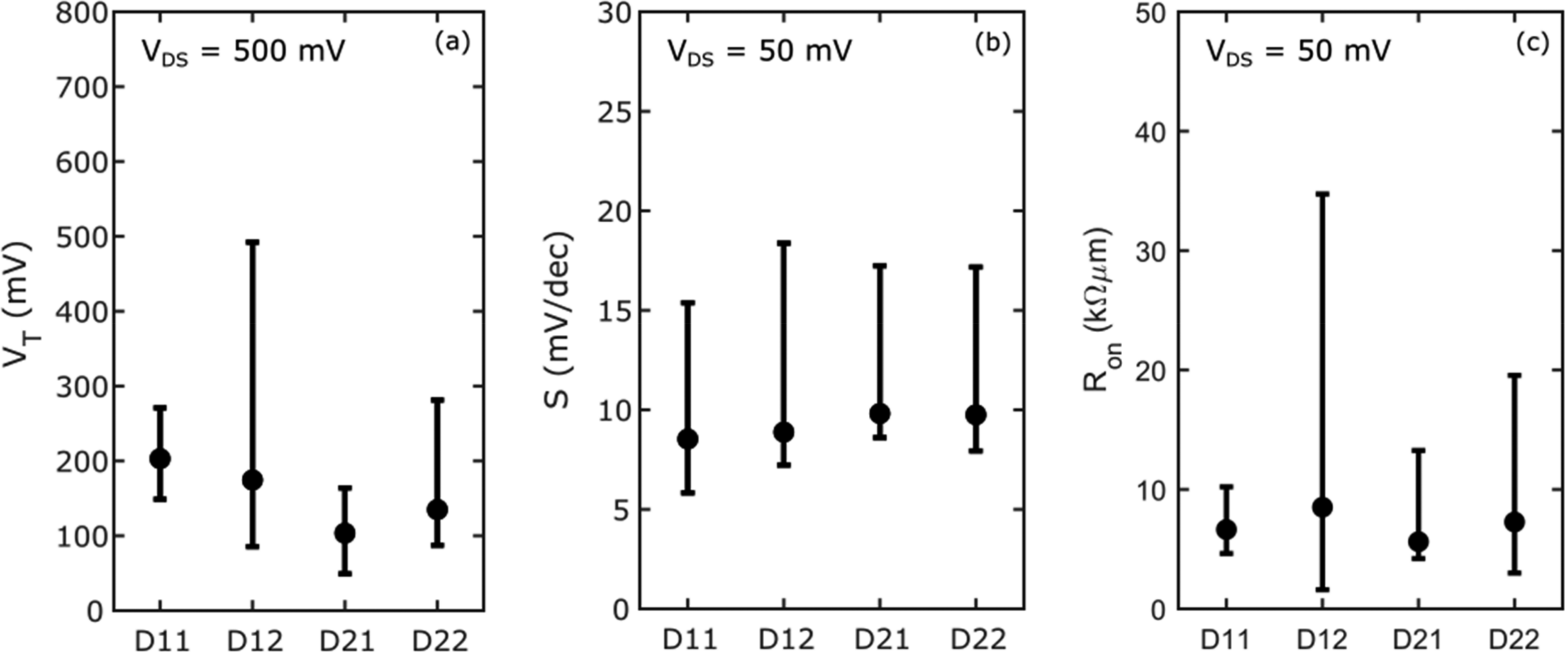
Extracted (a) threshold voltage, (b) minimum inverse subthreshold slope, and (c) on-resistance for different drain connections, measured on eight multiplexers at 13 K. The filled circles are the median value of each drain, and the bars show the 95% confidence interval.

Figure 5a compares the transfer characteristics at 13 K and 40 mK of the D_11_ current. For this specific drain, the minimum inverse subthreshold slope is 130, 6, and 2 mV/dec at 300 K, 13 K, and 40 mK, respectively. At cryogenic temperatures, the source/drain thermal energy is extremely sharp (*kT* = 3 μeV at 40 mK), potential fluctuations leading to 1D resonant tunneling type of behavior will lead to transmission resonances, which can enhance the subthreshold slope even for very low temperatures. This is in contrast to 2D type of devices, where averaging will lead to current limited by exponential band tails.^14−16^ At cryogenic temperatures the inverse subthreshold slope is probably limited by a combination of tunneling through potential fluctuations and interface trap states, while at room temperature limit is set by the Fermi–Dirac distribution and interface trap states. The very small inverse subthreshold slope is encouraging for low voltage operation in order to minimize the power dissipation for cryogenic operation, where cooling power often is very limited.

**Figure 5:**
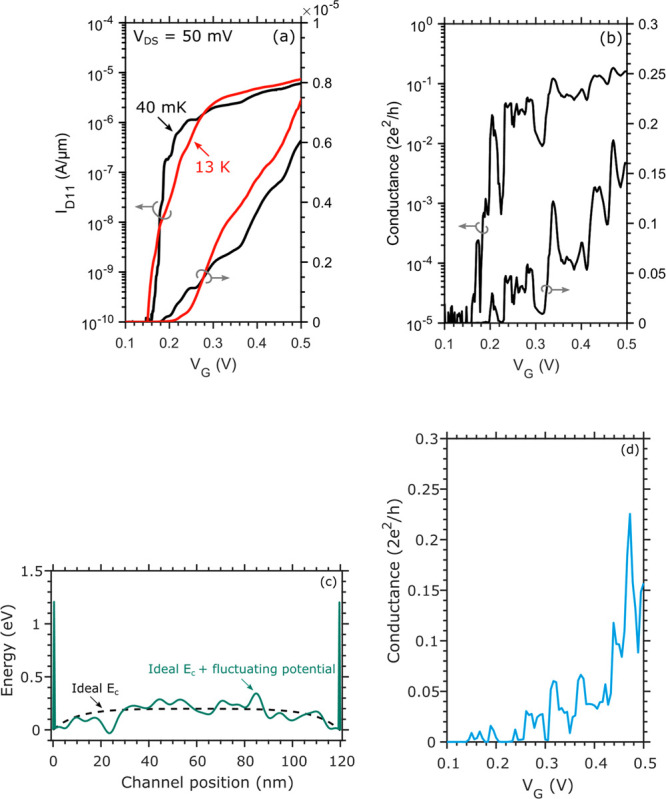
(a) Transfer characteristics normalized to the nanowire width of D_11_ with *V*_DS_ = 50 mV at 13 K and 40 mK. (b) Normalized conductance of D_11_ measured with *V*_DS_ = 5 mV at 40 mK. (c) Electrostatic potential along the channel used for calculations. A fluctuating potential with σ = 100 meV and *L* = 5 nm is superimposed on the ideal conduction band edge profile. (d) Simulated conductance characteristics at 40 mK using a 1D real space tight-binding nonequilibrium Green’s functions model.

Figure 5b shows the normalized conductance of D_11_ measured at 40 mK and a low *V*_DS_ = 5 mV, when sweeping gate B_1_. This is the minimum limit of the conductance since there is some voltage drop over the channel under gate A_1_ as well. At this relatively small bias window, several clear peaks and valleys appear in the conductance. We attribute this to charged defects in the oxide, changing the energy landscape and roughening the conduction band edge. This can be seen as quantum dots connected in series along the channel, which leads to resonance peaks in the transmission at certain energy levels. The resulting electron mobility degradation can be alleviated by moving the conducting channel away from the oxide interface by inserting a thin layer of InP.^17^ The drawback of this approach is the reduction of electrostatic control of the channel due to a lower gate-channel capacitance.

Presented in Figure 5c is such a fluctuating potential along the channel. A varying conduction band edge potential (mean standard deviation σ = 100 meV with a correlation length *L* = 5 nm) is superimposed on a smoothly varying background, obtained from an analytical solution of Poisson’s equation and 1D electrostatics. To calculate the transmission and hence the conduction through such a potential, a simple model based on a 1D real space tight-binding nonequilibrium Green’s functions (NEGF) model has been used.^18^ Each subband is added by an energy separation given by a two-band **k**·**p** model.^19^ The current and transmission through the fluctuating potential are then calculated using NEGF, the resulting conductance is presented in Figure 5d. This model can qualitatively explain the data, since the transmission and resonance peaks highly depend on the exact potential variation along the channel. In order to agree with the experimental data, high potential barriers (1.2 eV) are also needed to be added at the source-drain region. This indicates a need to optimize the regrown contact interface to enable a more transparent contact. This can potentially be achieved by additional cleaning prior to growth or further optimization of the growth parameters. The potential fluctuations within the channel can be minimized by reducing the charged defect concentration at the semiconductor/oxide interface.

In conclusion, we have designed and fabricated a 1-to-4 demultiplexer proof of concept device with good yield based on a selective area grown nanowire network. The design has been characterized at both 13 K and 40 mK and shows good isolation between gates and drains with very small gate leakage. The device exhibits a low minimum inverse subthreshold slope of 6 and 2 mV/dec at 13 K and 40 mK, respectively. At very low temperatures and low bias voltages, the transmission breaks up into resonance peaks due to a rough conduction band edge, highlighting the importance of oxide trap minimization especially when designing electronics for low-temperature operation.
